# The Effect of Overweight/Obesity on Cutaneous Microvascular Reactivity as Measured by Laser-Doppler Fluxmetry: A Systematic Review

**DOI:** 10.3390/biomedicines12112488

**Published:** 2024-10-30

**Authors:** Ally McIllhatton, Sean Lanting, Vivienne Chuter

**Affiliations:** 1 Discipline of Podiatry, College of Health, Medicine and Wellbeing, University of Newcastle, Ourimbah, NSW 2258, Australia; 2 Discipline of Podiatric Medicine, School of Health Sciences, Western Sydney University, Campbelltown, NSW 2560, Australia

**Keywords:** obesity, microvascular, laser-doppler, review

## Abstract

Introduction: We sought to determine by systematic review the independent effect of overweight/obesity on cutaneous microvascular reactivity in adults as measured by laser-Doppler fluxmetry. Methods: CINAHL Complete, SPORTSDiscus, Embase, Medline, and Cochrane Library were searched until March 2024 to identify studies investigating cutaneous microvascular reactivity in an overweight/obese but otherwise healthy group versus a lean/healthy weight. Reporting is consistent with the Preferred Reporting Items for Systematic Reviews and Meta-Analyses (PRISMA) statement. Quality appraisal of included studies was performed using the Strengthening the Reporting of Observational Studies in Epidemiology (STROBE) checklist. Results: Nineteen eligible articles reported on 1847 participants. Most articles reported impaired cutaneous microvascular reactivity in cohorts with overweight/obesity compared to cohorts with lean/healthy weight. Investigating reactivity via post-occlusive reactive hyperaemia (PORH) and iontophoresis of acetylcholine (ACh) has shown significance. No significant differences were reported between groups in response to local heating or to iontophoresis of methacholine or insulin, while findings of the effect of obesity on iontophoresis of sodium nitroprusside (SNP) were mixed. Conclusions: The pathophysiology of impaired cutaneous microvascular reactivity in overweight/obesity requires further investigation; however, impaired function of vasoactive substances, endothelial dysfunction, sensory nerves, and calcium-activated potassium channels may be implicated. Identifying these impaired microvascular responses should inform possible therapy targets in overweight and obesity.activated potassium channels may be implicated. Identifying these impaired microvascular responses should inform possible therapy targets in overweight and obesity.

## 1. Introduction

Overweight and obesity are defined as the accumulation of excessive fat due to extreme energy intake and low energy expenditure for prolonged periods of time [1,2]. The global increase of overweight and obesity has led to increased morbidity and mortality and is linked to widespread endothelial dysfunction, including macrovascular and microvascular disease [3,4]. The Australian Institute of Health and Welfare reported that in 2018 overweight and obesity were the second-leading contributors to fatal burden and are linked to at least 30 diseases, including cardiovascular disease, musculoskeletal conditions, type 2 diabetes, chronic kidney disease, and some cancers [4]. Further, obesity has been shown to specifically exacerbate microvascular dysfunction of multiple organ systems in the human body, leading to sequelae such as coronary, renal, and pulmonary dysfunction [5]. The aetiology of obesity-related microvascular dysfunction appears to be embedded in a combination of factors such as capillary rarefaction, endothelial dysfunction, the proliferation of pro-inflammatory adipokines and cytokines, and oxidative stress [6].

The skin provides an essential role in temperature regulation, tactile sensation, synthesis of vitamin D, provision of a physical barrier to infection, and in the facilitation of timely wound healing [7]. Cutaneous microvascular dysfunction has further implications for the regulation of hydrostatic pressure fluctuation, exchange of oxygen, nutrients, and hormones, as well as the determination of peripheral resistance [8,9]. Cutaneous microvascular reactivity involves examining the skin’s microvascular response to stimulus. A multitude of microvascular measures are available; however, laser-Doppler fluxmetry is most suited as it is a non-invasive [10] and reliable [11] method to measure microvascular flow independently of macrovascular involvement [12]. Further, laser-Doppler fluxmetry can investigate both endothelial-dependent and independent responses [13]. Stimuli used in assessment of cutaneous microvascular reactivity such as local heating or post-occlusive reactive hyperaemia (PORH) are at least partly endothelium-dependent [14] and therefore a marker of endothelial dysfunction, whereas iontophoresis or microdialysis of various chemical compounds can isolate endothelial-dependent (e.g., acetylcholine [ACh]) and -independent (e.g., sodium nitroprusside [SNP]) responses [8]. Several studies have investigated cutaneous microvascular reactivity in cohorts affected by overweight or obesity [15,16,17]. However, obesity is often associated with other contributors to microvascular dysfunction, such as type 2 diabetes [18]. Consequently, the independent effect of obesity on cutaneous microvascular reactivity requires clarification. A review of the effect of overweight/obesity on the microvascular function of multiple organ systems has previously been performed [5]. However, a review of the relationship with cutaneous microvascular reactivity is lacking. Our aim is to determine, by systematic review, the effect of overweight/obesity on cutaneous microvascular reactivity in adults as measured by laser-Doppler fluxmetry.

## 2. Methods

### 2.1. Eligibility Criteria

A variety of studies using laser-Doppler fluxmetry to investigate cutaneous microvascular reactivity in an adult group with overweight/obesity but otherwise healthy and an adult group with lean/healthy weight were included. Studies varied in design and were included if data of the specified cohorts and outcome measures were able to be extracted. Validated measures of body composition were required to determine the status of the specified groups (e.g., dual X-ray absorptiometry-derived measures of body composition or body mass index [BMI]). Studies were ineligible if they investigated microvascular function in non-cutaneous vascular beds (e.g., retinal or cardiac); used measures that reflected both micro and macrovascular function (e.g., transcutaneous oxygen pressure) or measures that did not investigate microvascular flow (e.g., capillaroscopy, peripheral arterial tonometry).

### 2.2. Search Strategy

**Figure 1:**
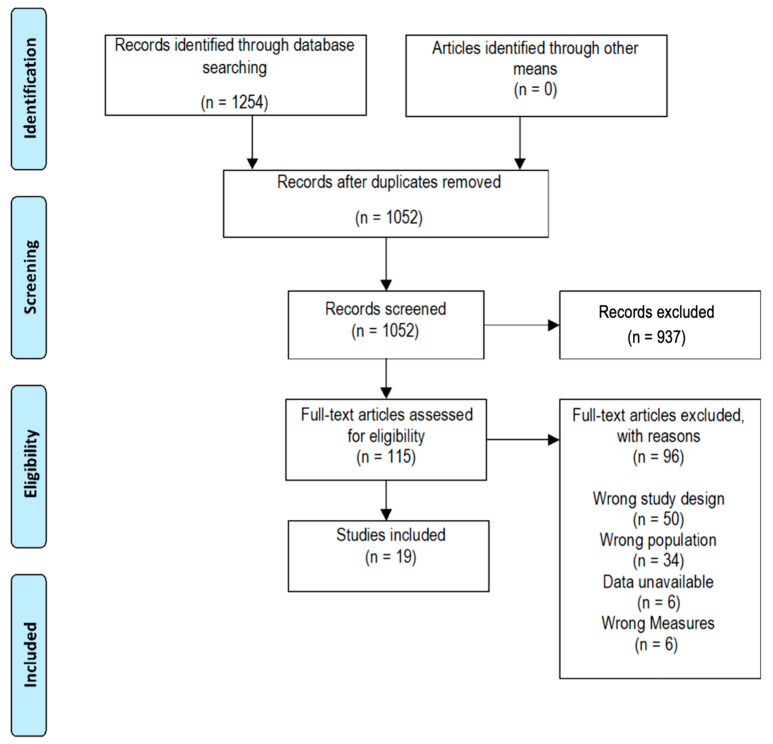
PRISMA flowchart.

This review was registered prospectively in the International Prospective Register of Systematic Reviews (PROSPERO ID: CRD42021289329), and reporting is consistent with the Preferred Reporting Items for Systematic Reviews and Meta-Analyses (PRISMA) statement (Figure 1) [19]. Electronic search was undertaken by one author (SL) using the biomedical databases CINAHL Complete, SPORTSDiscus, Embase, Medline, and Cochrane Library, including all articles, until March 2024 (Table 1). Retrieved citations were uploaded and managed through Covidence (www.covidence.org/). Independent screening of title, abstract, and full text was performed by two authors (AM and SL), and the final determination of study eligibility was performed in consultation with a third author (VC). Reference lists of both included articles and of review articles identified in the search were screened to identify any additionally relevant articles.

### 2.3. Assessment of Methodological Quality 

The Strengthening the Reporting of Observational Studies in Epidemiology (STROBE) tool [20] was used to assess the methodological quality and risk of bias of included articles (Appendix A). As the STROBE checklist is used to assess observational studies broadly, only items applicable to included studies were used. Quality appraisal was performed independently by two reviewers (AM and SL). Instances of disagreement or uncertainty were discussed, and arbitration by a third reviewer (VC) was not required. 

Overall risk of bias was interpreted from the STROBE checklist according to a completeness of reporting (COR) score, which was calculated as a percentage, where COR score (%) = [yes/(yes + no)] × 100 [21]. COR scores were assessed from an adaptation of the STROBE checklist to provide a quantitative measure, where <65% = unsatisfactory, 65–75% = fair, 76–85% = good, >85% = excellent [22].

### 2.4. Data Extraction 

Data were extracted by one author (AM) using a customised data extraction spreadsheet, which included article title, authors, year of publication, microvascular stimuli used (e.g., ACh iontophoresis), and location of stimuli application (e.g., forearm). To enable evaluation of the relationship between body composition and cutaneous microvascular reactivity, the following data were extracted for overweight/obese and lean groups: number of participants, age, sex, body composition measures (e.g., BMI), and results of cutaneous microvascular reactivity (e.g., peak response to stimulus). All data were then cross-checked by another author (SL). 

### 2.5. Statistical Analyses 

Data were prepared using Microsoft Excel and analysed with RevMan. An overview of included studies is provided using descriptive analyses. It was decided a priori that meta-analyses of the effect of overweight/obesity on cutaneous microvascular reactivity would be conducted, if possible. Given the expectation for a high degree of study heterogeneity, it was considered that a fixed effect meta-analysis would generally not be appropriate, and so we aimed to only pool estimates using a random effects approach if studies were sufficiently homogenous. The I^2^ statistic was used to assess heterogeneity of the models. Heterogeneity was interpreted as per the Cochrane guidelines: low (25%), moderate (50%), and high (75%) [23]. The importance of heterogeneity was based on more recent Cochrane guidelines: 0% to 40% (might not be important); 30% to 60% (may represent moderate heterogeneity); 50% to 90% (may represent substantial heterogeneity); and 75% to 100% (considerable heterogeneity) [24]. 

It was intended that sub-analyses of endothelial-dependent (e.g., Ach iontophoresis, local heating) and endothelial-independent (e.g., SNP iontophoresis) dysfunction, or combined (e.g., PORH), would be conducted where possible. 

## 3. Results 

Electronic database searches yielded 1254 studies for screening. Following application of eligibility criteria at title and abstract screening, 115 required full-text review. Nineteen studies met eligibility criteria for inclusion. 

### 3.1. Overview of Included Articles 

The 19 included studies investigated cutaneous microvascular reactivity in 1847 participants (Table 2). Mean age ranged from 21.88 to 62.0 years. Reported sex of participants was variable. All participants were reported to be normotensive with an absence of metabolic disease. Two studies reported the use of tobacco [25,26]. Overweight/obese groups were defined as BMI > 25 [25,26,27,28,29,30,31] or BMI > 35 [27,29,31,32,33,34,35,36,37,38,39,40,41,42,43]. Lean/healthy weight groups were defined in all studies as BMI < 25 [25,27,28,29,30,31,32,33,34,35,36,37,38,39,40,41,42,43]. BMI was used in all studies as a measure of body composition; however, additionally reported measures included body weight [25,26,30,31,32,33,34,37,41,43], waist circumference [25,26,28,29,31,32,35,38,39,40], hip circumference [29,31,32,39,40], waist–hip ratio [29,31,32,33,34,35,37], waist–height ratio [29], percentage body fat [25,26,30], visceral fat [25], fat mass [39,43], and fat-free mass [30,39]. Nine studies reported multiple outcomes with endothelial-dependent vasodilation as well as endothelial-independent vasodilation [30,32,33,37,41] or mixed origin measures [25,28,35,36]. Cutaneous microvascular reactivity as measured by laser-Doppler fluxmetry, with 16 studies [25,26,27,28,29,30,33,34,35,36,37,38,39,40,41,42] specifically using Periflux systems [44], was elicited by PORH [26,27,31,38,39,42,43], local heating [25,28,35,36] or by iontophoresis of ACh [25,26,28,29,32,33,35,36,37,40], methacholine (MCh) [30,41], insulin [34], and/or SNP [26,30,32,33,37,41]. All microvascular measures were obtained in the upper limb with the forearm dominantly used [25,26,28,29,30,31,32,34,35,36,38,39,40,41,42], followed by fingers [27,31,33,37].

### 3.2. The Effect of Overweight/Obesity on Cutaneous Microvascular Reactivity 

In response to iontophoresis with ACh [28,29,32,33,35,37,40] and PORH [27,31,38,39,42], seven and five studies, respectively, reported statistically significant findings indicating impairment of cutaneous microvascular reactivity in the upper limbs of groups affected by overweight and obesity (Table 3).

However, three studies reported non-significant findings in response to PORH [26,38,43] and four studies in response to iontophoresis with ACh [25,26,29,36]. Six studies investigating the response of iontophoresis with SNP reported mixed findings [26,30,32,33,37,41]. All studies investigating cutaneous microvascular reactivity in groups affected by overweight/obesity through iontophoresis with MCh [30,41], insulin [34], and local heating [25,28,36] reported non-significant findings.

### 3.3. Methodological Quality 

All studies provided appropriate outcomes, confounding factors, adjusted estimates, and discussed analyses, interactions of participants, and potential sources of bias (Appendix A). Four studies described setting, recruitment, exposures, and data collection comprehensively [25,26,38,42]. Eleven studies failed to describe how the study size was arrived at [27,28,29,32,34,35,36,39,40,41,43]. Three studies reported the numbers of individuals at each stage of the study and gave reasons for non-participation [25,26,42], and two studies did not discuss the limitations of the study or the magnitude of any bias [27,28]. The mean COR score was reported as 86% across included articles, indicating an “excellent” score [22].

Outcome measures (endothelial-dependent, endothelial-independent, and mixed origin measures) were individually explored for meta-analysis. However, heterogeneity was found to be excessively high for each independent measure (i.e., I^2^ > 75%). Differences between studies, including anatomical site of measurement, sex ratio of participants, and outcome measure protocols, likely contributed to the high heterogeneity.

## 4. Discussion

The aim of this review was to systematically evaluate the literature investigating cutaneous microvascular reactivity as measured by laser-Doppler fluxmetry in otherwise healthy groups with overweight/obesity compared with lean/healthy weight counterparts. This review supports that overweight and obesity have a significant effect on microvascular reactivity compared to a healthy weight, suggesting there may be a reduction in endothelium-dependent and mixed responses in people with overweight/obesity via iontophoresis of ACh and PORH. However, this finding was not strongly replicated for endothelium independent responses. The extent to which these findings can be interpreted must be considered in the context of the high heterogeneity between studies, which prevents meta-analysis of these data.

### 4.1. Endothelial-Dependent Vasodilation

Included studies used a variety of protocols to investigate endothelial-dependent vasodilation in cutaneous microvasculature. Iontophoresis with ACh was a widely used measure of cutaneous microvascular reactivity. Studies using iontophoresis and ACh that reported no statistically significant difference between groups are suggested to be due to a small sample size, an all-female cohort, and a young age possibly contributing to a lower duration of overweight/obesity [25,26,29,36]. Though a useful measure, there is some conflicting evidence as to the exact mechanism of ACh iontophoresis vasodilation [45,46]. For example, it has been suggested that this response is a COX-dependent pathway and that nitric-oxide does not extensively contribute [47,48,49,50]. Other research indicates that the vasodilatory effect is due to activated nitric-oxide synthase and prostaglandin production [51], causing a release of nitric oxide resulting in a cascade of biological events relaxing smooth muscle cells [48]. Nonetheless, this review highlights that cutaneous vasodilation instigated by ACh iontophoresis appears to be blunted in the presence of obesity to a statistically significant extent.

To reduce the potential hydrolysing effects of acetylcholinesterase occurring in iontophoresis of ACh [52] and to address concerns regarding inter-site and inter-day variability [53], two studies performed iontophoresis of MCh [30,41]. This stimulus acts directly on muscarinic receptors and, unlike other vasodilators, potentiates insulin-mediated glucose uptake by muscle [54]. In these studies, no significant cutaneous microvascular impairment was found between groups affected by overweight/obesity compared with groups of a lean/healthy weight. It has been suggested that attenuated nitric-oxide vasodilation may result in compensation from prostaglandins or endothelial-derived hyperpolarising factors. This may have contributed to the non-significant findings in these two studies involving cohorts of a younger age contributing to a lower duration of overweight/obesity [30,41].

Insulin has been shown to have a similar vasodilatory effect on skin microvasculature to ACh as it stimulates nitric-oxide synthase and increases the release of nitric-oxide from vascular endothelium [55,56]. Only one study investigated the response to both insulin and a control substance in groups affected by overweight/obesity and groups of a lean/healthy weight [34]. While groups of a lean/healthy weight demonstrated a significant difference in response to insulin compared to the control substance (*p* = 0.04), the groups affected by overweight/obesity did not (*p* = 0.7) [34]. An earlier study by De Jongh [33], although it had a smaller sample size, still showed significance with the use of iontophoresis and ACh in similar demographic and identical methods apart from the drug used during iontophoresis [33], suggesting iontophoresis of insulin may not be an effective method. This has been seen in iontophoresis delivery of insulin, which has proven challenging due to the low permeability through skin due to a high molecular weight; therefore, the absolute amount of insulin delivered at the level of the microvasculature is uncertain [57,58].

### 4.2. Mixed-Origin Vasodilation

#### 4.2.1. Post-Occlusive Reactive Hyperaemia

Five of seven studies reported impairment of reactivity with PORH in cohorts with overweight/obesity versus lean/healthy weight [27,31,38,39,42]. The lack of significant difference between groups in some studies may be attributed to the relatively small sample size [26,43], short duration of obesity [26], absence of measure in visceral fat [43], and/or due to investigating an exclusively female cohort [43]. While PORH has historically been linked to the COX pathway [59], robust research suggests a greater role of sensory nerves and large-conductance calcium-activated potassium channels [60]. Coupled with the findings regarding ACh iontophoresis, there are likely to be multiple contributing pathways to obesity-related cutaneous microvascular dysfunction.

#### 4.2.2. Local Heating

Three studies investigated cutaneous microvascular reactivity in response to local heating [25,28,36]. This vasodilatory stimulus appears to have a complex, mixed mechanism, including for initial peak response and sustained plateau response. For instance, while nitric-oxide release likely plays a role, evidence suggests local sensory nerves and neurotransmitters such as norepinephrine and neuropeptide Y also play a critical role in inducing vasodilation [61]. While questions remain regarding this mechanism, the lack of significant difference between groups affected by overweight/obesity compared with groups of a lean/healthy weight in response to local heating suggests that the origin of obesity-related cutaneous microvascular dysfunction may not be overwhelmingly influenced by these neurological elements. Given the role of sensory nerves to PORH and consistent impairment in populations with obesity to this stimuli, further research is required to provide distinction of this mechanism.

### 4.3. Endothelial-Independent Vasodilation

Endothelial-independent vasodilation directly affects vascular smooth muscle cells and is mediated by substances such as nitric-oxide donors, including SNP. Previous research suggests that endothelial-independent vasodilation is largely preserved in obesity, indicating that the primary defect is at the level of the endothelium rather than at the smooth muscle in animal models [62,63]; however, research is limited in human studies.

This may contribute to a lack of significant findings in studies investigating SNP iontophoresis vasodilation [26,32,33]. Two studies reported that participants with obesity and those of a healthy weight both achieved maximal cutaneous vascular conductance (CVC) responses following the final dose of SNP [30,41]. However, a significantly higher dose of SNP was required to elicit 50% of the maximal CVC response in the participants with overweight/obesity compared to lean/healthy weight groups [30,41]. This indicates modification of nitric-oxide action on the microvasculature leading to an attenuation of vascular smooth muscle function. Additionally, Patik and Christmas [41] disclosed that insulin sensitivity, waist circumference, or total body fat were not considered, which may affect microvascular function as the central-adiposity phenotype has been linked to vascular complications. Further, studies reporting alternate findings lead to inconsistency, making it difficult to comprehensively determine the effect or otherwise of overweight/obesity on endothelium-dependent cutaneous microvascular function, and this requires further investigation.

### 4.4. Body Composition

Most included studies used BMI as the primary measure to categorise participants into an obese, overweight, or healthy weight group. BMI has been strongly associated with percentage of body fat [64,65] and is a globally accepted method of classifying overweight and obesity [66]. However, BMI does not provide information pertaining to muscle mass or distribution of adipose tissue, leading to limitations in its capacity to predict proportion/amount of body fat, specifically due to variations in body composition associated with age, sex, and ethnicity [66,67,68]. It is widely discussed that BMI does not provide an accurate representation of an individual’s body composition as it only captures data relating to height and weight. Studies have indicated that type and distribution of adipose tissue, specifically visceral adipose tissue, is a strong predictor of health risks; however, the overall amount of adipose tissue is not [69,70]. This suggests additional measures can provide insight into whether the participant is at a higher risk of cardiovascular complications. All studies included in this review reported additional anthropometric data; however, due to an absence of analyses indicating the accuracy of BMI per participant involved, there is not a clear understanding of the allocation of participants into overweight/obese or healthy weight groups beyond the BMI information provided.

### 4.5. Future Directions

Regardless of the pathophysiology of obesity-related blunted cutaneous microvascular reactivity, research should focus on amelioration of this dysfunction to prevent adverse health effects. Exercise therapies such as endurance, moderate-intensity aerobic, and high-intensity interval training have demonstrated macrovascular benefits in people affected by obesity [71]. However, while meta-analysis has demonstrated improved cutaneous microvascular reactivity in healthy older adults with a moderate effect size, following moderate-intensity aerobic training [72], further research is needed to explore the extent this benefit may apply in the presence of obesity. Further consideration of adipose tissue distribution is required to better understand the role this may play in microvascular function as well as the duration of overweight/obesity to determine at what point microvascular dysfunction may become evident. Additionally, pharmacotherapies such as antihypertensives [73] and dipeptidyl peptidase IV inhibitors [74] have shown promise in improving microvascular function in similar cohorts. Further research is required to establish the precise mechanism of obesity-related cutaneous microvascular dysfunction and to identify potential target therapies such as other pharmacotherapies, risk factor modification, and lifestyle interventions.

### 4.6. Limitations

Articles in this review only investigated cutaneous microvascular reactivity in the upper limbs, and it is unclear if findings are applicable to the lower limb or indeed to other vascular beds such as cardiac or pulmonary. Secondly, we only included studies that employed laser-Doppler fluxmetry to determine cutaneous microvascular reactivity, and further clinical knowledge could be obtained by reviewing other peripheral vascular measures with slightly varied measures such as capillaroscopy or transcutaneous oxygen pressure (TcPO_2_). Additionally, despite the relatively high number of included studies, significant methodological differences resulted in large heterogeneity and, as such, precluded meta-analysis. Furthermore, while quality appraisal found included articles to be methodologically sound, there are some questions regarding reproducibility of some measures [75], and therefore a degree of caution is warranted when interpreting the findings. STROBE guidelines do not have a commonly used quantitative measure to score observational studies, which may result in differences in assessing the quality of the publications. Lastly, somewhat mixed findings of primary outcomes between studies and sometimes contradictory understanding of mechanisms of cutaneous microvascular reactivity (e.g., endothelial-dependent, -independent, or mixed) further complicate the overall findings of this review.

## 5. Conclusions

Obesity is often complicated by other vascular risk factors, such as diabetes and other components of metabolic syndrome. Most studies included in this review reported microvascular reactivity is impaired in the upper limbs of groups affected by overweight/obesity compared with groups of a lean/healthy weight, as determined by laser-Doppler fluxmetry. Preclusion of meta-analysis due to heterogeneity of included studies prevented the extent and significance of this relationship being determined. The mechanism of impairment likely involves smooth muscle cells, endothelial dysfunction, and sensory nerves, though further research is required to elaborate on this pathophysiology. While further research considerations are needed in this area, impaired cutaneous microvascular responses to PORH as well as to iontophoresis of ACh offer promising insight to inform possible therapy targets.

## Figures and Tables

**Table 1:** Search strategy.

Search 1	Overweight or obes* or body fat* or adipos* or body mass index or bmi or body weight or weight or body composition or waist circumference or waist-hip or waist to hip or abdominal fat or intra-abdominal fat or subcutaneous or visceral
Search 2	Reactive hyperaemia or reactive hyperemia or cutaneous reactivity or skin reactivity or post-occlusive or postocclusive or post-ischemi* or post-ischaemi* or PORH or PRH or iontophoresis or thermal hyperaemia or thermal hyperemia or local heating or microdialysis
Search 3	Laser-doppler or laser doppler or laser doppler fluxmetry or laser doppler flowmetry or microvascular or microangiopathy
Search 4	Search 1 AND Search 2 AND Search 3

**Table 2:** Participant Characteristics—age is reported as mean ± SD, median (IQR), or range. Overweight/obese cohorts are considered obese (i.e., BMI > 35) unless otherwise stated. NR, not reported.

	Lean				Overweight/Obese			
Authors	Participant No.	Age (Years)	Sex (% Male)	Smoking Status (%)	Participant No.	Age (Years)	Sex (% Male)	Smoking Status (%)
Al-Tahami et al. (2011) [32]	36	26.54 ± 0.60	22	0	36	26.58 ± 0.89	22	0
Czernichow et al. (2010) [25]	130	62.0 ± 5.9	45	13.1	120	61.9 ± 5.6	51	5.8
De Jongh et al. (2004) [33]	16	38.9 ± 6.7	0	0	12	38.8 ± 7.0	0	0
De Jongh et al. (2008) [34]	40	39.1 ± 9.4	0	0	40	40.1 ± 6.6	0	0
Dimassi et al. (2016) [35]	46	33.11 ± 1.46	39	0	69	36.46 ± 1.36	39	0
Dimassi et al. (2018) [36]	6	22.33 ± 1.47	0	0	9	21.88 ± 0.99	0	0
Escobar et al. (2023) [26]	32	33 ± 10	40	0	24	34 ± 5	46	2
Jonk et al. (2011) [37]	20	32.0 (25.3–44.0)	35	0	19	35.0 (23.0–54.0)	26	0
Kilic et al. (2022) [27]	28	43.1 ± 8.4	NR	NR	25	48.2 ± 9.7	NR	NR
Korolev et al. (2020) [31]	15	47 (38–49)	100	NR	Overweight: 21 Obese: 7	Overweight: 45 (40–49) Obese: 49 (43–51)	100	NR
Martin-Rodriguez et al. (2014) [38]	30	37 ± 11	27	NR	23	40 ± 9	17	NR
Mastantuono et al. (2016) [39]	54	(55–64)	50	0	54	55–64	50	0
Miadi-Messaoud et al. (2010) [28]	60	30.43 ± 1.38	0	0	Overweight: 50 Obese: 70	Overweight: 31.58 ± 1.77 Obese: 37.21 ± 1.35	0	0
Nasr et al. (2016) [40]	211	40.7 ± 11.9	48	0	183	41.9 ± 11.5	44	0
Patik et al. (2016) [41]	14	24.7 ± 4.7	64	0	15	23.4 ± 4.6	53	0
Rossi et al. (2011) [42]	28	44 ± 10	25	NR	19	40 ± 8	75	NR
Seywert et al. (2004) [43]	8	24.6 ± 3.5	0	0	8	27.8 ± 5.1	100	0
Touir et al. (2021) [29]	186	NR	NR	0	Overweight: 35 Obese: 84	NR	NR	0
Tucker et al. (2018) [30]	10	24 ± 4	100	NR	10	26 ± 5	0	NR

**Table 3:** Cutaneous microvascular reactivity in lean vs. overweight/obese cohorts—cutaneous microvascular reactivity in response to various stimuli.

Author	Equipment	Site	Drug	Method	Outcome Measure	Results Lean	Results Overweight/Obese	*p*-Value
Al-Tahami et al. (2011) [32]	Dual-channel DRT4 laser Doppler	Right forearm	ACh powder diluted in sodium chloride 0.9%	0.4 mL of drug solution in ACh chamber attached to anodal lead. Two minutes of baseline perfusion followed by 5 current pulses (0.007 mA for 2 min) separated by 1 min of current free intervals.	Peak (AU)	71.03 ± 7.13	40.45 ± 6.59	0.001
Czernichow et al. (2010) [25]	Periflux System 5000	Forearm	ACh chloride 2% solution (800 μL)	Skin heated to 33 °C. ACh chamber attached to anodal lead. Four minutes of baseline perfusion followed by 3 doses (10 mA for 10 s) separated by 2 min of current free intervals.	P%BL	416 ± 32	478 ± 38	0.193
De Jongh et al. (2004) [33]	Periflux System 4000	Dorsum of middle phalanx of 3rd finger	ACh 1%	ACh chamber attached to anodal lead delivered 9 doses (0.1 mA for 20 s) separated by 1 min of current free intervals.	Plateau (PU) % increase	154.2 ± 48.9 537 ± 133	106.4 ± 40.9 345 ± 159	<0.05 <0.05
Dimassi et al. (2016) [35]	Periflux System 5000	Forearm	ACh 2%	Skin heated to 32 °C. ACh chamber attached to anodal lead. Two minutes of baseline perfusion followed by 3 doses (0.1 mA for 10 s) separated by 2 min of current free intervals.	CVC_max_ (PU/mmHg) CVC_max_—BL (PU/mmHg)	0.47 ± 0.05 0.39 ± 0.04	0.32 ± 0.03 0.27 ± 0.03	0.014 0.033
Dimassi et al. (2018) [36]	Periflux System 5000	Forearm	ACh 2%	Skin heated tomin 32 °C. ACh chamber attached to anodal lead. Two minutes of baseline perfusion followed by 3 doses (0.1 mA for 10 s) separated by 2 min of current free intervals.	CVC_max_ (PU/mmHg)	0.18 ± 0.05	0.15 ± 0.06	>0.05
Jonk et al. (2011) [37]	Periflux System 5000	Middle phalanx of 2nd finger, left hand	ACh 1%	Skin heated to 32 °C. ACh chamber attached to anodal lead that delivered seven doses (0.1 mA for 20 s) separated by 1 min of current free intervals.	Plateau (PU)	92.3 ± 55.2	71.1 ± 46.6	<0.05
Miadi-Messaoud et al. (2010) [28]	Periflux System 5000	Forearm	ACh 2% (200 μL)	Skin heated to 33 °C. ACh chamber attached to anodal lead. Four minutes of baseline perfusion followed by 4 doses at 10 mA for 10 s (16, 32, 48 and 96 µg mL^−1^) separated by 2 min of current free intervals.	P%BL	1167.97 ± 77.59	Overweight: 643.34 ± 38.17 Obese: 323.39 ± 18.31	<0.0001
Nasr et al. (2016) [40]	Periflux System 5000	Forearm	Ach 2% (200 μL)	Skin heated to 33 °C. Ach chamber attached to anodal lead. Four min of baseline perfusion followed by 4 doses at 10 mA for 10 s (16, 32, 48 and 96 µg mL^−1^) separated by 2 min of current free intervals.	P%BL	469 (190–1260)	271 (47–985)	0.012
Touir et al. (2021) [29]	Periflux System 5000	Forearm	ACh chloride 2% solution	Skin heated to 32 °C. ACh chamber attached to anodal lead. Two minutes of baseline perfusion followed by 3 doses (0.1 mA for 10 s) separated by 2 min of current free intervals.	(i) CVC_max_ (PU/mmHg) (ii) CVC_max_—BL (PU/mmHg)	57 ± 0.04 0.42 ± 0.04	Overweight: 0.42 ± 0.08 0.4 ± 0.08	>0.05 >0.05
							Obese: 0.24 ± 0.03 0.19 ± 0.03	0.002 <0.05
**Iontophoresis (SNP)**								
**Author**	**Equipment**	**Site**	**Drug**	**Method**	**Outcome Measure**	**Results Lean**	**Results Overweight/Obese**	***p*** **-Value**
Al-Tahami et al. (2011) [32]	Dual-channel DRT4 laser Doppler	Right forearm	SNP powder diluted in sodium chloride 0.9%	0.4 mL of drug solution in SNP chamber attached to cathodal lead. Two minutes of baseline perfusion followed by 5 current pulses (0.007 mA for 2 min) separated by 1 min of current free intervals.	Peak (AU)	74.43 ± 9.28	50.24 ± 7.37	0.053
De Jongh et al. (2004) [33]	Periflux System 4000	Dorsum of middle phalanx of 3rd finger	SNP 0.01%	SNP chamber attached to cathodal lead delivered 7 doses (0.2 mA for 20 s) separated by 3 min of current free intervals.	(i) Plateau (PU) (ii) % increase	160.9 ±48.9 476 ± 186	132.1 ± 57.3 471 ± 301	>0.05 >0.05
Jonk et al. (2011) [37]	Periflux System 5000	Middle phalanx of 4th finger, left hand	SNP 0.01%	Skin maintained above 30 °C. SNP chamber attached to cathodal lead delivered 9 doses (0.2 mA for 20 s) separated by 90 s of current free intervals.	Plateau (PU)	147.9 ± 83.1	117.7 ± 71.2	<0.05
Patik et al. (2016) [41]	Periflux System 5000	Dorsum of non-dominant forearm	SNP diluted in Ringer’s solution	Skin heated to 33 °C. SNP chamber attached to cathodal lead. Ten minutes of baseline perfusion followed by 7 doses (ranging 5 × 10^−8^ to 5 × 10^−2^ M) for 8 min at 0.2 μL. The final dose was infused for 20 min to establish a plateau in LDF response	(i) CVC_max_ (mmHg^−1^) (ii) %CVC_max_ (EC_50_)	3.3 ± 0.2 −3.746	2.6 ± 0.2 −2.931	0.11 <0.001
Tucker et al. (2018) [30]	Periflux System 5000	Dorsum of left forearm	SNP	A baseline measure was recorded followed by SNP perfusion starting at a concentration of 5 × 10^−8^ M increasing tenfold to a max dose of 5 × 10^−2^ M for one min each at a rate of 100 µL min^−1^ before being switched to a rate of 4 µL min^−1^ for an additional 4 min so that each dose was administered for at least 5 min	(i) %CVC_max_ (ii) %CVC_max_ (EC_50_)	48.8 ± 6.8 −2.13 ± 0.06	44.8 ± 9.6 −1.74 ± 0.17	0.729 0.034
**Iontophoresis (Insulin)**								
**Author**	**Equipment**	**Site**	**Drug**	**Method**	**Outcome Measure**	**Results Lean**	**Results Overweight/Obese**	***p*** **-Value**
De Jongh et al. (2008) [34]	Periflux System 4000	Dorsal side of non-dominant wrist	Insulin (0.20 mL Velosulin 100 IE/mL) and diluting medium	Skin maintained above 30 °C. Insulin chamber attached to cathodal lead. One minute of baseline perfusion followed by 12 doses (0.2 mA for 20 s) separated by 90 s of current free intervals	(i) Peak (PU) (ii) P%BL	31.6 (17.1–43.9) 205 ± 58	28.1 (14.4–47.1) 95 ± 23	NR NR
**Iontophoresis (MCh)**								
**Author**	**Equipment**	**Site**	**Drug**	**Method**	**Outcome Measure**	**Results Lean**	**Results Overweight/Obese**	***p*** **-Value**
Patik et al. (2016) [41]	Periflux System 5000	Dorsum of non-dominant forearm	Acetyl-β-methacholine chloride diluted in Ringer’s solution	Skin heated to 33 °C. MCh chamber attached to anodal lead. Ten minutes of baseline perfusion followed by 7 doses (ranging 10−6 to 1 M) for 8 min at 0.2 μL. The final dose was infused for 20 min to establish a plateau in LDF response	(i) CVC_max_ (mmHg^−1^) (ii) %CVC_max_ (EC_50_)	3.3 ± 0.6 −3.852 ± 0.25	2.9 ± 0.5 −3.796 ±0.23	0.13 0.81
Tucker et al. (2018) [30]	Periflux System 5000	Dorsum of left forearm	MCh chloride	A baseline measure was recorded followed by MCh perfusion starting at a concentration of 1 × 10^−7^ M increasing tenfold to a max dose of 1 × 10^−1^ M at a rate of 100 µL min^−1^ before being switched to a rate of 4 µL min^−1^ for an additional 4 min so that each dose was administered for at least 5 min	(i) %CVC_max_ (ii) %CVC_max_ (EC_50_)	76.4 ± 5.9 −3.04 ± 0.11	82.0 ± 7.6 −2.98 ± 0.19	0.568 0.841
**Post-Occlusive Reactive Hyperaemia (PORH)**								
**Author**	**Equipment**	**Site**		**Method**	**Outcome Measure**	**Results Lean**	**Results Overweight/Obese**	***p*** **-Value**
Kilic et al. (2022) [27]	Periflux System 5000	Fourth finger, right hand		Baseline measured for 3 min followed by occlusion around finger inflated to 250 mmHg and held for 3 min and deflated.	Peak (PU)	514.5 ± 163.7	367.3 ± 132.3	0.002
Korolev et al. (2020) [31]	Two-channel laser analyser LAKK-02	Left forearm		Baseline measured for 10 min followed by occlusion inflated to 50 mmHg above systolic pressure and held for 5 min and then deflated.	P%BL	264% (Q25 = 234%, Q75 = 332%)	Overweight: 239% (Q25 = 195%, Q75 = 309%) Obese: 211% (Q25 = 163%, Q75 = 222%)	<0.05
		Left distal phalanx of 3rd finger			P%BL	130% (Q25 = 112%, Q75 = 187%)	Overweight: 110% (Q25 = 108%, Q75 = 125%) Obese: 109% (Q25 = 102%, Q75 = 123%)	<0.05
Martin-Rodriguez et al. (2014) [38]	Periflux System 5000	Forearm		Baseline measured for 3 min followed by occlusion around right arm inflated to 220 mmHg and held for 4 min and deflated. Flux recording continued for at least 5 min.	(i) CVC_max_ (mmHg^−1^) (ii) CVC_max_—BL (mmHg^−1^) (iii) AuC post-ischaemia (mmHg^−1^)	0.42 ± 0.1 0.35 ± 0.1 11.49 ± 4.6	0.40 ± 0.2 0.32 ± 0.2 8.64 ± 3.9	0.286 0.216 <0.001
Mastantuono et al. (2016) [39]	Periflux System 4001	Volar surface of right forearm		Baseline measured 20 min followed by occlusion around right arm at 50 mmHg above systolic pressure and held for 2 min and then deflated.	(i) Peak (PU) (ii) Time to Peak (seconds) (iii) P%BL	72.3 ± 1.5 7.1 ± 0.3 670 ± 15.6	60.3 ± 2.5 5.8 ± 0.2 552 ± 27.4	<0.01 <0.01 NR
Rossi et al. (2011) [42]	Periflux System 4001	Right forearm		Baseline measured for 15 min followed by occlusion around right arm for 3 min at 30 mmHg above systolic pressure and then deflated.	P%BL	611 ± 37	481 ± 62	0.013
Seywert et al. (2004) [43]	Laser Doppler Imager (moorLDI2-IR)	Forearm		Baseline measured followed by occlusion (220 mmHg) for 3 min.	(i) Peak (PU) (ii) AuC post-ischaemia (PU*min)	223 ± 85 100 ± 52	197 ± 60 117 ± 48	>0.05 >0.05
**Local Heating**								
**Author**	**Equipment**	**Site**		**Method**	**Outcome Measure**	**Results Lean**	**Results Overweight/Obese**	***p*-Value**
Czernichow et al. (2010) [25]	Periflux System 5000	Forearm		Skin temperature maintained at 33 °C and increased to 44 °C for 5 min.	P%BL (AU)	625 ± 36	713 ± 38	0.100
Dimassi et al. (2018) [36]	Periflux System 5000	Forearm		Skin temperature maintained at 32 °C and increased to 44 °C for 5 min.	CVC_max_ (PU/mmHg)	0.60 ± 0.19	0.72 ± 0.21	>0.05
Miadi-Messaoud et al. (2010) [28]	Periflux System 5000	Forearm		Skin temperature maintained at 32 °C and increased to 44 °C for 5 min.	P%BL (AU)	1425 ± 829	Overweight: 1428 ± 767 Obese: 1257 ± 731	>0.05

Values are expressed as mean ± SD unless indicated otherwise. Ach, acetylcholine; SNP, sodium nitroprusside; P%BL, peak response as a percentage of baseline; CVC, cutaneous vascular conductance; CVC_max_, maximum cutaneous vascular conductance; PU, perfusion units; NR, not reported; BL, baseline; AuC, area under the curve; MCh, methacholine; EC_50_, concentration of drug needed to elicit 50% of the maximal response. Specific terms have been used to group alike outcome measures terms used by individual articles: peak (ACh_max_, acetylcholine-mediated vasoilation, SNP_max_, peak flow, PORH_peak_ CVC, peak value, heating-mediated vasodilation); P%BL (FSBF_max_, FSBF%, percent increase from baseline, PORH(%), maximal response); % increase; CVC_max_ (peak ACh-CVC, CVC flux, peak LSH-CVC); CVC_max_-BL(ACh-CVC, LSH-CVC, PORH_max_ CVC); %CVC_max_; % CVC_max_ (EC_50_); AuC post-ischemia (AUC_O_ CVC, AUC (PU·min)).

## Data Availability

The authors confirm that the data supporting the findings of this study are available within the article.

## Abbreviations

PORH	Post-occlusive reactive hyperaemia
ACh	Acetylcholine
SNP	Sodium nitroprusside
BMI	Body mass index
PRISMA	Preferred Reporting Items for Systematic Reviews and Meta-Analyses
STROBE	Strengthening the Reporting of Observational Studies in Epidemiology
COR	Completeness of Reporting
SPSS	Statistical Package for the Social Sciences
MCh	Methacholine
TcP02	Transcutaneous oxygen pressure

## Appendix A

**Table A1:** Strengthening the reporting of observational studies in epidemiology.

Item		Al-Tahami, 2011 [32]	Czernichow, 2010 [25]	De Jongh, 2004 [33]	De Jongh, 2008 [34]	Dimassi, 2016 [35]	Dimassi, 2018 [36]	Jonk, 2011 [37]	Kilic, 2022 [27]	Korolev, 2020 [31]	Martin-Rodriguez, 2014 [38]	Mastantuono, 2016 [39]	Miadi-Messaoud, 2010 [28]	Nasr, 2016 [40]	Patik, 2016 [41]	Rossi, 2011 [42]	Seywert, 2004 [43]	Touir, 2021 [29]	Tucker, 2018 [30]
1(a)	Indicate the study’s design with a commonly used term in the title or the abstract	✓	✓	✗	✗	✓	✗	✗	✗	✗	✓	✗	✗	✓	✗	✓	✓	✗	✗
1(b)	Provide in the abstract an informative and balanced summary of what was done and what was found	✓	✓	✓	✓	✓	✓	✓	✓	✓	✓	✓	✓	✓	✓	✓	✓	✓	✓
2	Explain the scientific background and rationale for the investigation being reported	✓	✓	✓	✓	✓	✓	✓	✓	✓	✓	✓	✓	✓	✓	✓	✓	✓	✓
3	State specific objectives, including any prespecified hypotheses	✓	✓	✓	✓	✓	✓	✓	✓	✓	✓	✓	✓	✓	✓	✓	✓	✓	✓
4	Present key elements of study design early in the paper	✓	✓	✓	✓	✓	✓	✓	✓	✓	✓	✓	✓	✓	✓	✓	✓	✓	✓
5	Describe the setting, locations, and relevant dates, including periods of recruitment, exposure, follow-up, and data collection	✗	✓	✗	✗	✗	✗	✗	✗	✗	✓	✗	✗	✗	✗	✓	✗	✗	✗
6(a)	Give the eligibility criteria, and the sources and methods of case ascertainment and control selection. Give the rationale for the choice of cases and controls	✓	✓	✓	✓	✓	✓	✓	✓	✓	✓	✓	✓	✓	✓	✓	✓	✓	✓
6(b)	For matched studies, give matching criteria and the number of controls per case	✓	NA	NA	NA	✓	NA	NA	NA	NA	✓	✓	NA	NA	✓	NA	✓	NA	NA
7	Clearly define all outcomes, exposures, predictors, potential confounders, and effect modifiers. Give diagnostic criteria, if applicable	✓	✓	✓	✓	✓	✓	✓	✓	✓	✓	✓	✓	✓	✓	✓	✓	✓	✓
8	For each variable of interest, give sources of data and details of methods of assessment (measurement). Describe comparability of assessment methods if there is more than one group	✓	✓	✓	✓	✓	✓	✓	✓	✓	✓	✓	✓	✓	✓	✓	✓	✓	✓
9	Describe any efforts to address potential sources of bias	✓	✓	✓	✓	✓	✓	✓	✓	✓	✓	✓	✓	✓	✓	✓	✓	✓	✓
10	Explain how the study size was arrived at	✗	✓	✓	✗	✗	✗	✓	✗	✗	✓	✗	✗	✗	✗	✓	✗	✓	✓
11	Explain how quantitative variables were handled in the analyses. If applicable, describe which groupings were chosen and why	✓	✓	✓	✓	✓	✓	✓	✓	✓	✓	✓	✓	✓	✓	✓	✓	✓	✓
12(a)	Describe all statistical methods, including those used to control for confounding	✓	✓	✓	✓	✓	✓	✓	✓	✓	✓	✓	✓	✓	✓	✓	✓	✓	✓
12(b)	Describe any methods used to examine subgroups and interactions	NA	✓	✓	✓	✓	✓	✓	✓	✓	✓	✓	✓	✓	✓	✓	✓	✓	✓
12(c)	Explain how missing data were addressed	NA	✓	NA	NA	NA	NA	NA	NA	NA	NA	NA	NA	NA	✓	NA	NA	✓	NA
12(d)	If applicable, explain how matching of cases and controls was addressed	✓	✓	✓	NA	✓	NA	NA	✓	✓	✓	✗	NA	NA	✓	NA	NA	✓	NA
12(e)	Describe any sensitivity analyses	✓	✓	✓	✓	✓	✓	✓	✓	✓	✓	✓	✓	✓	✓	✓	✓	✓	✓
13(a)	Report numbers of individuals at each stage of study—e.g., numbers potentially eligible, examined for eligibility, confirmed eligible, included in the study, completing follow-up, and analysed	✗	✓	✗	✗	✗	✗	✗	✗	✗	✗	✗	✗	✗	✗	✓	✗	✗	✗
13(b)	Give reasons for non-participation at each stage	NA	✓	NA	NA	NA	NA	NA	NA	NA	NA	NA	NA	NA	NA	✓	NA	NA	NA
13(c)	Consider use of a flow diagram	✗	✗	✗	✗	✗	✗	✗	✗	✗	✗	✗	✗	✗	✗	✗	✗	✗	✗
14(a)	Give characteristics of study participants (e.g., demographic, clinical, social) and information on exposures and potential confounders	✓	✓	✓	✓	✓	✓	✓	✓	✓	✓	✓	✓	✓	✓	✓	✓	✓	✓
14(b)	Indicate number of participants with missing data for each variable of interest	NA	NA	NA	NA	NA	NA	NA	NA	NA	NA	NA	NA	NA	✓	NA	NA	NA	✓
15	Report numbers in each exposure category, or summary measures of exposure	✓	✓	✓	✓	✓	✓	✓	✓	✓	✓	✓	✓	✓	✓	✓	✓	✓	✓
16(a)	Give unadjusted estimates and, if applicable, confounder-adjusted estimates and their precision (e.g., 95% confidence interval). Make clear which confounders were adjusted for and why they were included	✓	✓	✓	✓	✓	✓	✓	✓	✓	✓	✓	✓	✓	✓	✓	✓	✓	✓
16(b)	Report category boundaries when continuous variables were categorized	✓	✓	✓	✓	✓	✓	✓	✓	✓	✓	✓	✓	✓	✓	✓	✓	✓	✓
16(c)	If relevant, consider translating estimates of relative risk into absolute risk for a meaningful time period	✗	✗	✗	✗	✗	✗	✗	✗	✗	✗	✗	✗	✓	✗	✗	✗	✗	✗
17	Report other analyses done—e.g., analyses of subgroups and interactions, and sensitivity analyses	✓	✓	✓	✓	✓	✓	✓	✓	✓	✓	✓	✓	✓	✓	✓	✓	✓	✓
18	Summarise key results with reference to study objectives	✓	✓	✓	✓	✓	✓	✓	✓	✓	✓	✓	✓	✓	✓	✓	✓	✓	✓
19	Discuss limitations of the study, taking into account sources of potential bias or imprecision. Discuss both direction and magnitude of any potential bias	✓	✓	✓	✓	✓	✓	✓	✗	✓	✓	✓	✗	✓	✓	✓	✓	✓	✓
20	Give a cautious overall interpretation of results considering objectives, limitations, multiplicity of analyses, results from similar studies, and other relevant evidence	✓	✓	✓	✓	✓	✓	✓	✓	✓	✓	✓	✓	✓	✓	✓	✓	✓	✓
21	Discuss the generalisability (external validity) of the study results	✓	✓	✓	✓	✓	✓	✓	✓	✓	✓	✓	✓	✓	✓	✓	✓	✓	✓
22	Give the source of funding and the role of the funders for the present study and, if applicable, for the original study on which the present article is based	✓	✓	✓	✓	✗	✗	✓	✓	✗	✓	✗	✗	✓	✓	✗	✗	✗	✓

✓: item is included, ✗: item is missing, NA: item is not applicable.
